# Acceptable outcomes with unicompartmental knee replacement and PCL deficiency are achievable: a case series of nine patients

**DOI:** 10.1007/s00167-020-06112-3

**Published:** 2020-07-08

**Authors:** Pengfei Li, James Kennedy, Hasan Raza Mohammad, Zhihui Pang, Stephen Mellon, William Jackson, Andrew Price, Christopher Dodd, David Murray

**Affiliations:** 1 Nuffield Department of Orthopaedics, Rheumatology and Musculoskeletal Sciences, University of Oxford, Oxford, OX3 7LD UK; 2 Jiangmen Central Hospital/Affiliated Jiangmen Hospital of Sun Yat-sen University, Jiangmen, 529030 China; 3 Department of Orthopaedics, The First Affiliated Hospital of Guangzhou University of Chinese Medicine, Guangzhou, 510405 China; 4 Nuffield Orthopaedic Centre, Oxford, OX3 7LD UK

**Keywords:** Posterior cruciate ligament, Oxford unicompartmental knee replacement, Functional outcome, Implant survival, Unicondylar knee replacement

## Abstract

**Purpose:**

Posterior cruciate ligament (PCL) deficiency is considered to be a contraindication for unicompartmental knee replacement (UKR); however, there is no evidence to support or contradict this. There are occasional circumstances where UKR in PCL deficient patients have been performed where the patient otherwise satisfies the indications for UKR. The aim of this paper is to describe the outcome of UKR in PCL deficient patients.

**Methods:**

A retrospective study of patients with painful medial compartment osteoarthritis and PCL deficiency treated with Oxford UKR between 2006 and 2015 was undertaken. Clinical records from a prospectively recorded database were reviewed and outcomes were assessed based on revision rate, Oxford Knee Score (OKS), American Knee Society score and Tegner Activity Score.

**Results:**

Nine patients were identified. The median age at surgery was 51 years (range 42–80) and median follow-up was 6 years (range 1–10). There was one bearing dislocation requiring open exchange. The outcome of seven patients was excellent (OKS > 41). Two patients, who were both elderly, had good outcomes (OKS 41 and 39). One patient had a poor outcome, but it is not clear if this was related to the knee as she had a learning disability and examination and radiographs of the knee were satisfactory.

**Conclusion:**

The results of this small series suggest that excellent results can be achieved with UKR for selected patients with medial osteoarthritis in a PCL deficient knee that was functioning well before the osteoarthritis developed. On the basis of this a larger study should be undertaken. Until more results are available PCL deficiency should be considered a relative contra-indication to UKR.

**Level of evidence:**

IV.

## Introduction

Unicompartmental knee replacement (UKR) is an effective treatment for knee osteoarthritis. Compared to total knee replacement (TKR), UKR has a faster recovery, lower morbidity and mortality, better functional outcomes, and is more cost effective [1, 2, 9, 19]. One of the primary aims of UKR is to restore knee kinematics to normal. This is only possible if all the ligaments are functionally intact, even if they are not anatomically normal. The common situation in which there is knee osteoarthritis with functionally normal ligaments is anteromedial osteoarthritis [17]. Therefore the main indication for UKR is anteromedial osteoarthritis with bone-onbone medially, full thickness cartilage laterally and functionally normal ligaments. These criteria are satisfied in about half of patients needing knee replacement [5, 16, 18].

As the ligaments need to be functionally normal to achieve normal kinematics after UKR, posterior cruciate ligament (PCL) deficiency is considered to be a contra-indication [4]. However, this is not evidence based and the role of PCL deficiency in UKR has not previously been studied. Isolated PCL injuries are rare, but after the injury patients often function well until they develop painful medial osteoarthritis. In the author’s practice, particularly if there are mitigating circumstance, UKR has occasionally been performed in PCL deficient knees providing the patient otherwise meets the indications for the procedure. The aim of this study was to review the functional and revision outcomes of the few patients with medial osteoarthritis and PCL deficiency who have been treated with UKR. The study hypothesis was that these patients would have an unacceptably high revision rate with poor functional outcomes.

## Materials and methods

A retrospective case series is described of patients with medial osteoarthritis and PCL deficiency who were treated with Oxford UKR by four surgeons. Apart from the PCL deficiency the patients satisfied the indications for UKR, meaning that they had bone-on-bone medial osteoarthritis, functionally intact medial collateral and anterior cruciate ligaments, and full thickness cartilage in the lateral compartment. Age, activity, obesity, chondral ulcers on the medial side of the lateral femoral condyle and the state of the patellofemoral joint (unless there was lateral bone loss, grooving and subluxation) were ignored [6, 7, 15].

**Fig. 1:**
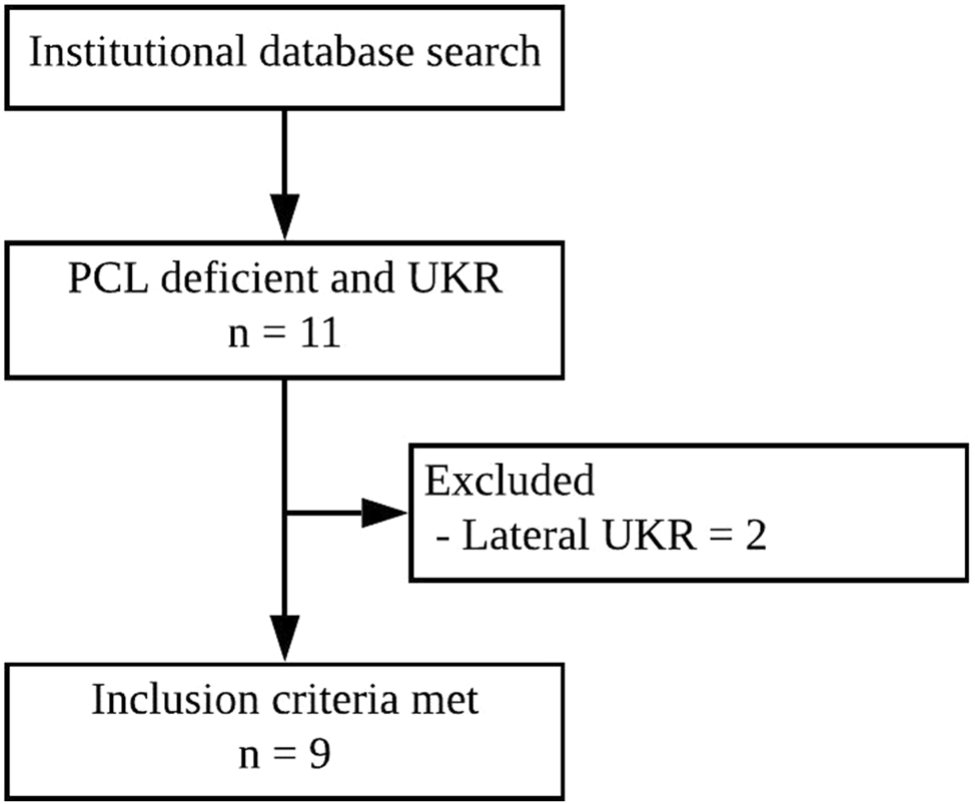
Patient flow chart. *PCL* posterior cruciate ligament, *UKR* unicompartmental knee replacement

Patients were identified from a prospectively recorded database of UKR, and were included if they had PCL deficiency and a medial UKR (Fig. 1). Patients were excluded if they had other ligament deficiency (e.g., anterior cruciate ligament), osteotomy, or other UKR procedures such as a lateral UKR or patellofemoral replacement. Where PCL deficiency was recorded, the patients’ case notes, operation records and clinic letters were reviewed to determine the reason for their PCL deficiency and why they had been offered a UKR. Patients were assessed pre-operatively and at 1, 2 and 5 years using a standard protocol of clinical review with functional assessment. Assessments were made by research physiotherapists, independent of the surgical and clinical teams involved in the patients’ care. Functional outcomes were assessed using the Oxford Knee Score (with OKS > 41 considered excellent), the American Knee Society objective and functional (AKSS-O and AKSS-F) Score, and the Tegner Activity score. Implant survival was determined with the endpoint being revision, defined as exchange, removal or addition of any component. In addition complications and re-operations were recorded. In the normal knee at 90° of flexion, the tibial tuberosity should lie about 1 cm forward from the distal femoral condyles. The degree of PCL laxity was graded, if done, by using the posterior drawer test intraoperatively. Grade 1 was considered as > 0.5 cm laxity relative to the contralateral knee, Grade 2 if the anterior tibia could be translated posteriorly to the femoral condyles (indicating > 1 cm of posterior translation), and in Grade 3 the anterior tibia could be translated posteriorly to the distal femoral condyles [3].

Ethical approval was sought from the local research ethics committee and deemed not required, as information was collected as part of routine patient care.

## Results

Between 2006 and 2015, 9 patients underwent Phase 3 Oxford UKR (Zimmer Biomet, Bridgend, United Kingdom) with a minimally invasive approach for medial osteoarthritis and PCL deficiency. Two were cemented and seven cementless. The median age was 51 (range 42–80) and the median follow-up was 6 years (range 1–10). The outcome of six patients was excellent (OKS > 41), while that of 1 patient was poor (OKS 22). There has been one revision in which a new bearing was inserted for a dislocation, occurring 9 years after the primary surgery. This knee had previously suffered a bearing dislocation that was successfully reduced by a manipulation under anaesthetic at 18 months. There were no serious medical complications but one patient had severe post-operative constipation. The characteristics and outcome scores are shown in Tables 1 and 2. A brief description of each case is given below.

**Table 1:** Patient characteristics at surgery

	Year of surgery	Side	Age at surgery (years)	Previous injury	Correctable Varus	PCL laxity^b^	Fixation
Patient 1	2009	Left	43	Yes	5°	Grade 1	Cementless
Patient 2	2009	Right	43	Yes	^a^	^a^	Cemented
Patient 3	2010	Left	61	Yes	^a^	^a^	Cementless
Patient 4	2010	Right	55	Yes	^a^	^a^	Cemented
Patient 5	2011	Left	45	No	15°	^a^	Cementless
Patient 6	2011	Right	46	Yes	^a^	Grade 2	Cementless
Patient 7	2012	Right	80	No	^a^	Grade 2	Cementless
Patient 8	2015	Right	51	Yes	^a^	Grade 2	Cementless
Patient 9	2015	Right	75	No	5°	^a^	Cementless

*PCL* posterior cruciate ligament

^a^Data missing

^b^Grade 1: 0.5–1 cm laxity, Grade 2: tibia can be translated to femoral condyles, Grade 3: tibia can be translated beyond the femoral condyles

**Table 2:** Functional scores and complications

	Follow up (years)	Pre-operative score				Score at latest follow up				Complication
		OKS	Tegner	AKSS-0	AKSS-F	OKS	Tegner	AKSS-0	AKSS-F	
Patient 1	10	28	3	50	90	47	3	80	100	Dislocation
Patient 2	3	28	3	47	90	42	3	84	100	Instability
Patient 3	9	24	^a^	50	70	46	6	90	100	None
Patient 4	8	37	3	75	70	47	3	95	100	None
Patient 5	1	^a^	^a^	^a^	^a^	22	1	^a^	75	Poor OKS
Patient 6	6	^a^	^a^	^a^	^a^	48	5	^a^	100	None
Patient 7	7	35	3	85	80	39	3	95	80	None
Patient 8	4	34	4	79	100	48	5	^a^	100	None
Patient 9	2	27	3	70	90	41	3	95	70	None

*OKS* Oxford Knee Score, *Tegner* Tegner Activity Score, *AKSS-O and AKSS-F* American Knee Society Score Objective and Functional component respectively

^a^Data missing

*Patient 1* A 43-year-old male who sustained a PCL injury whilst running when 20 years-old, subsequently developed bone-on-bone medial compartment osteoarthritis. 18 months following cementless UKR he sustained a dislocation of the bearing when crouching. He underwent a manipulation under anaesthetic and the bearing was felt to reduce, which was confirmed with fluoroscopy. Eight years later he sustained another dislocation and underwent an open bearing exchange. Whilst the bearing showed signs of anterior wear suggesting anterior impingement, a definite cause could not be found. All the components were found to be well-fixed, and a new 4 mm bearing was inserted without complication. At 10 years follow-up, the outcome was excellent (OKS 47).

*Patient 2* A 43-year-old female who presented with night pain, instability and activity limitation. She had sustained a knee injury in her late 20s whilst playing football, however, it was only at arthroscopic assessment at another centre that she was found to be PCL deficient. Following cemented UKR she was satisfied and pain free on daily activities. However, she continued to have some instability approximately three times a week and anterior knee pain. Despite this, at 3 year follow-up, her OKS was 42 so her outcome was considered excellent.

*Patient 3* A 61-year-old male presented with pain and severe medial compartment osteoarthritis. He had sustained a previous PCL injury associated with a tibial fracture. Nine years following the cementless UKR the outcome was excellent (OKS 46).

*Patient 4* A 55-year-old male presented with knee pain affecting his golfing, and was found to have severe medial osteoarthritis. When he was young he had sustained an injury to the knee which had caused a PCL disruption and meniscal damage which was treated with an open meniscectomy. Eight years following cemented UKR, the outcome was excellent (OKS 47).

*Patient 5* A 45-year-old female whose symptoms had deteriorated significantly with pain at night and a dramatically reduced walking distance. She had a learning disability necessitating a residential carer and was overweight. She had a complicated course with severe post-operative constipation. One year following cementless UKR, her OKS was 22, which is categorised as poor. However her range of motion was 0°–120° and the X-ray was satisfactory.

*Patient 6* A 46-year-old male who injured his knee when young in a road traffic accident and sustained a PCL injury. He was restricted in his activities by pain. 6 years following the cementless UKR, the outcome was excellent (OKS 48).

*Patient 7* An 80-year-old male with PCL deficiency but no definite history of previous major knee trauma presented with significant pain. Seven years following the cementless UKR the outcome was good (OKS 39).

*Patient 8* A 51-year-old male presented with pain particularly over the medial aspect of the joint and could not kneel, which was a problem as he was a builder. He had injured his knee skiing 7 years previously. Four years following cementless UKR, the outcome was excellent (OKS 48).

**Fig. 2:**
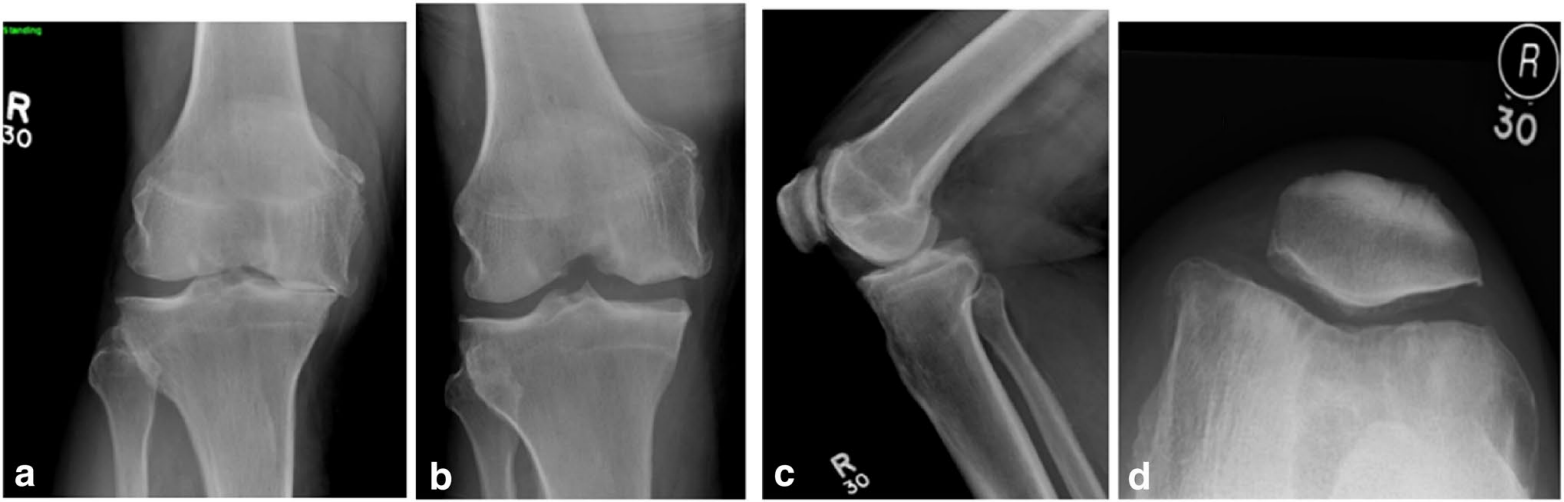
X-ray showed severe medial compartment arthritis with bone loss. **a** Rosenberg at 30° flexion, **b** Valgus stress, **c** lateral, **d** Skyline

**Fig. 3:**
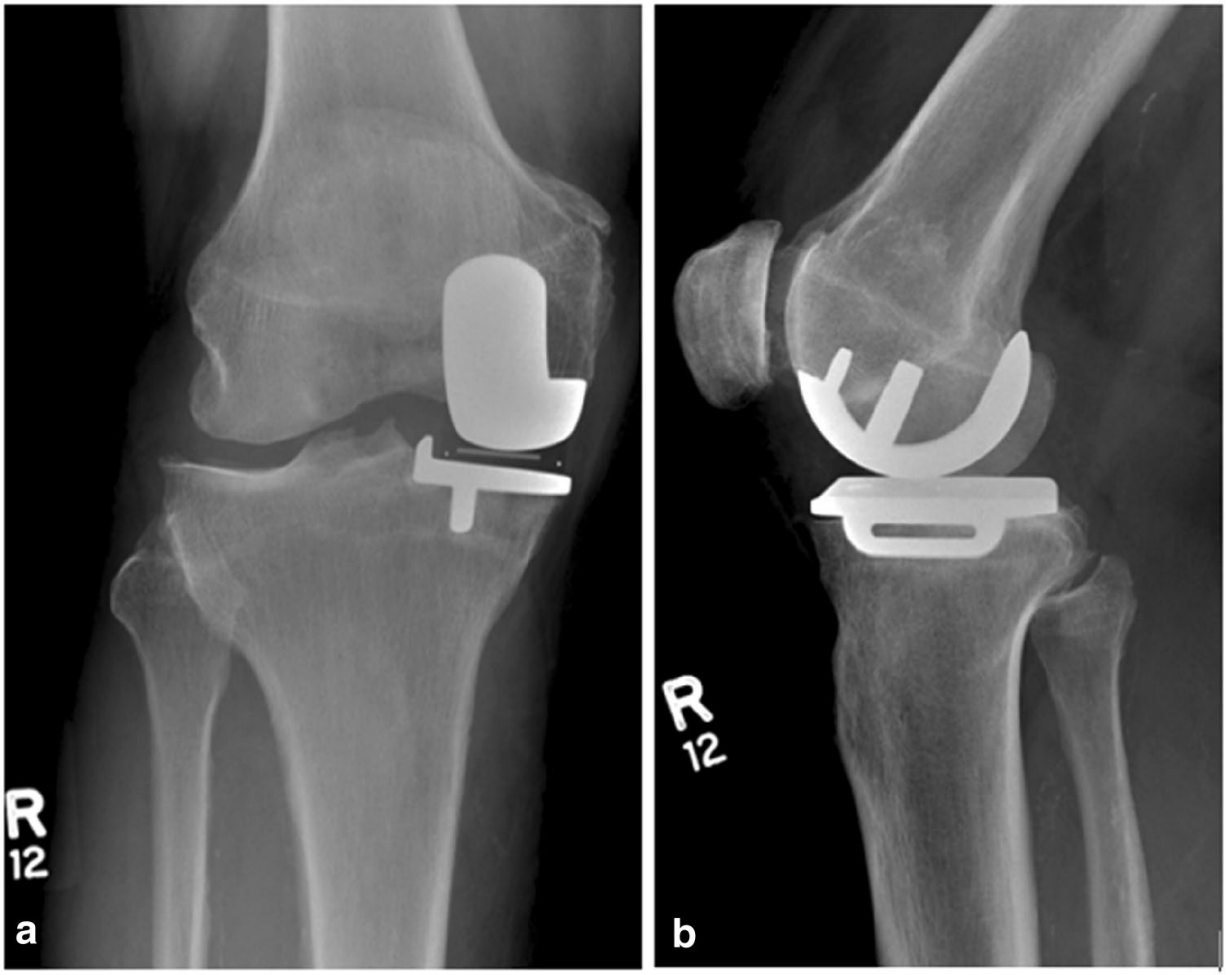
X-Ray of same patient as Fig. 2, showing cementless medial UKR in situ

*Patient 9* A 75-year-old male presented with pain in his knee such that he could not go up and down stairs normally and walking was limited. X-ray showed severe medial compartment osteoarthritis with bone loss (Fig. 2). He had a significant medical history including pulmonary embolus, coronary artery bypass and spinal problems. He had evidence of PCL deficiency at operation and underwent cementless Oxford UKR (Fig. 3). At 2 year follow-up, the outcome was good, with an OKS of 41.

## Discussion

This case series demonstrates that the results of Oxford UKR used for medial compartment osteoarthritis in knees with PCL deficiency tend to be very good. However, as the numbers are small, the follow up short and the indications poorly defined no firm recommendations can be made, other than that further study is needed. As the results are encouraging it would be not unreasonable to undertake more UKR in these patients to try and define the indications more precisely and determine which groups will benefit.

The majority of patients (5/9) in the study were young, in their 40s and 50s, fit and active. These patients often have disappointing result with TKR. They find that their activities are limited and they have a high revision rate [13]. The decision to implant a UKR was made mainly to address these issues. It was hoped that if a UKR was implanted the functional results would be better and the TKR would be delayed. In all cases, the patients were warned that in their situation the procedure should be considered as experimental. The results were, however, remarkably good. In all the young fit patients the OKS was considered excellent with a mean score of 46 (range 42–48). Their activity levels were also high with a mean Tegner score of 4.2 (range 3–6). None have required revision and, from the appearance of their radiographs, it is unlikely that they will require a revision in the near future. One of these patients did have a somewhat disappointing result in that, even though her OKS was 42, she complained of some instability approximately three times a week and anterior knee pain. One of her original complaints was instability, so perhaps significant instability should be considered to be a relative contra-indication to UKR for medial osteoarthritis with PCL deficiency. Therefore, it would seem sensible to continue implanting UKR in young active patients with painful medial osteoarthritis and PCL deficiency, who had good function before developing osteoarthritis, as this seems to be a good indicator of their post-operative performance.

The other main subgroup of patients (3/9) that had UKR for medial osteoarthritis with PCL deficiency, were those who it was thought would benefit from the minimally invasive nature of the procedure, which compared to TKR provides a faster recovery with few complications and lower morbidly and mortality [19]. Two of the patients were elderly (age 75 and 80 at the time of surgery) and the younger of these had significant medical co-morbidities. The third patient, although young, was overweight and had a learning disability. The two elderly patients had OKS of 39 and 41, which are considered good. However the OKS tends to deteriorate with increasing age so scores of this level in these patients who were aged 87 and 77 at follow up should probably be considered to be excellent. The third patient did not do so well but it is not clear if the problems were related to the knee replacement, as clinical examination and radiographs suggested a well-functioning knee. The results would therefore suggest that it is sensible to continue to do UKR with PCL deficiency in the elderly, particularly if they are unfit.

There was only one patient in the intermediate age group, which contains the majority of patients having knee replacement, and presumably the majority of patients with medial osteoarthritis and PCL deficiency needing a knee replacement. This patient, who was aged 61, had an excellent result (OKS 46, Tegner 6). As both younger and older patients did well, it would be not unreasonable to do UKR with PCL deficiency in this intermediate group as well, so as to determine how well it performs. These patients should not only benefit from the improved function of the UKR but also the faster recovery and lower morbidity and mortality.

The PCL is the largest and strongest intra-articular knee ligament. It is an essential passive stabiliser of the knee joint, serving as the primary restraint to excessive posterior tibial translation and a significant constraint to internal rotation beyond 90° of flexion [8, 12]. However, the PCL is not loaded appreciably during activities of daily living during flexion [11]. PCL injury usually occurs in the context of multiligament knee injuries, but isolated injury does occur and results in altered joint kinematics and can occasionally lead to instability [10]. Following PCL injury, in the medial compartment there is posterior subluxation of the medial tibial plateau with the femoral condyle articulating with the upsloping anterior portion of the medial tibial joint surface which can lead to medial osteoarthritis [10]. Even in young athletes with well-functioning PCL-deficient knees, early medial cartilage degeneration can be detected on MRI [14]. In contrast in the lateral compartment knee kinematics are not altered by PCL rupture so lateral osteoarthritis tends not to occur [10]. These findings help to explain the results of this study. In particular they explain why painful medial osteoarthritis occurs in otherwise well-functioning knees following isolated PCL injury, and why after UKR, which treats the pain, good function is likely to occur. They also explain why lateral osteoarthritis is unlikely to occur and hopefully why it will not occur after medial UKR. However, after UKR with PCL deficiency, the kinematics and therefore loading in the medial compartment will be abnormal so the long term outcome remains unknown.

This study is important as it is the first report of the outcome of UKR in PCL deficient knees. The major weakness is the small number of patients. This is because isolated PCL injury is rare and, although PCL deficiency leads to an increased rate of osteoarthritis, the number of PCL deficient patients that could be treated with UKR is very small. In the authors’ practice UKR implanted in PCL deficient knees is substantially less than 1% of all UKR. The other main weakness is the short period of follow up. However, on the basis of the encouraging results in this study, the authors, and, hopefully, other high volume UKR surgeons, will consider doing more cases so hopefully in the future there will be larger studies with longer follow-up on which recommendations can be based. Until then PCL deficiency should be considered to be a relative contra-indication to UKR.
